# Age and cancer type: associations with increased odds of receiving a late diagnosis in people with advanced cancer

**DOI:** 10.1186/s12885-023-11652-1

**Published:** 2023-11-30

**Authors:** Sarah Mills, Peter Donnan, Deans Buchanan, Blair H. Smith

**Affiliations:** 1 Population and Behavioural Science Division, School of Medicine, University of St Andrews, North Haugh, St Andrews, KY16 9T Scotland; 2 Population Health and Genomics Division, University of Dundee Medical School Mackenzie Building, Ninewells Hospital and Medical School, Kirsty Semple Way, Dundee, DD2 4BF Scotland; 3 NHS Tayside, Ninewells Hospital, South Block, Level 7, Dundee, DD2 4BF Scotland

**Keywords:** Cancer, Delayed diagnosis, Palliative care

## Abstract

**Purpose:**

In order to deliver appropriate and timely care planning and minimise avoidable late diagnoses, clinicians need to be aware of which patients are at higher risk of receiving a late cancer diagnosis. We aimed to determine which demographic and clinical factors are associated with receiving a ‘late’ cancer diagnosis (within the last 12 weeks of life).

**Method:**

Retrospective cohort study of 2,443 people who died from cancer (‘cancer decedents’) in 2013–2015. Demographic and cancer registry datasets linked using patient-identifying Community Health Index numbers. Analysis used binary logistic regression, with univariate and adjusted odds ratios (SPSS v25).

**Results:**

One third (*n* = 831,34.0%) received a late diagnosis. Age and cancer type were significantly associated with late cancer diagnosis (*p* < 0.001). Other demographic factors were not associated with receiving a late diagnosis. Cancer decedents with lung cancer (Odds Ratios presented in abstract are the inverse of those presented in the main text, where lung cancer is the reference category. Presented as 1/(OR multivariate)) were more likely to have late diagnosis than those with bowel (95% Confidence Interval [95%CI] Odds Ratio (OR)1.52 (OR1.12 to 2.04)), breast or ovarian (95%CI OR3.33 (OR2.27 to 5.0) or prostate (95%CI OR9.09 (OR4.0 to 20.0)) cancers. Cancer decedents aged > 85 years had higher odds of late diagnosis (95%CI OR3.45 (OR2.63 to 4.55)), compared to those aged < 65 years.

**Conclusions:**

Cancer decedents who were older and those with lung cancer were significantly more likely to receive late cancer diagnoses than those who were younger or who had other cancer types.

**Supplementary Information:**

The online version contains supplementary material available at 10.1186/s12885-023-11652-1.

## Key messages

• One third (*n*=831,34.0%) of cancer decedents had late diagnoses (within the last 12 weeks of life).

• Cancer decedents with lung cancer had higher odds of late diagnosis than those with other cancers.

• Cancer decedents >85 years old were 3 times more likely to have late diagnoses than those <65 years old.

**Supplementary Information:**

The online version contains supplementary material available at 10.1186/s12885-023-11652-1.

## Introduction

Cancer accounts for one in three deaths in the UK, with the number of people dying from cancer increasing annually [1–3]. Despite improvements in cancer survivability, the UK has some of the worse cancer outcomes in Europe, with late cancer diagnoses playing a substantial role in these poor outcomes [4–6]. Late cancer diagnosis confers a substantial, and potentially avoidable, excess morbidity and mortality, compared to cancer diagnosed at an earlier stage [7, 8].

Identifying which patients are at greatest risk of receiving a late diagnosis will allow clinicians and policymakers to target resources to those for whom they will confer the greatest benefit. For the purposes of this research, the authors have defined ‘late diagnosis’ as a cancer diagnosis occurring within twelve weeks of a patient’s date of death.

Receiving a late diagnosis denies patients dying from cancer the opportunity to receive good quality palliative care and timely anticipatory care planning, as well as time for putting affairs in order, spending time with loves ones, and coming to terms with their diagnosis. Among patients who die from cancer, those who received late diagnoses were less likely to be prescribed strong opioids and anticipatory palliative care medication, compared to those who were diagnosed earlier [9]. In the UK, most people who die from cancer wish to die at home and avoid unnecessarily aggressive treatment or hospitalisation at the end of their lives [10–14]. Patients with late diagnoses are less likely to die in their preferred place of death and are more likely to be frequent users of unscheduled and emergency care, compared to those who did not have late diagnoses [15–19]. In addition to the benefits in survivorship and quality of life, minimising late cancer diagnoses has a substantial economic benefit. Cancer Research UK (CRUK) estimates that, in England alone, reducing avoidable late diagnoses would improve the survival chances of 52,000 people and save the NHS over £210 million annually in treatment costs [4].

Exploring factors related to late diagnosis has become even more important since the advent of the Covid-19 pandemic, which resulted in a 42% reduction in the number of patients whose cancer has been diagnosed through screening, and a 12% reduction in people starting cancer treatment, compared to pre-pandemic levels [20]. The UK’s National Health Service (NHS) is expecting a surge in late diagnoses as those people with cancer who were missed by screening, and not referred through early primary care access, are identified later in their disease course [7, 21, 22].

Illness-related behaviour, including presenting to healthcare services, may be influenced by demographic factors; however, few studies have addressed the relative impact of each demographic factors independently, in order to identify which ones have the greatest association with receiving a late diagnosis. This study aims to identify any associations between demographic factor and cancer type, with receiving a late diagnosis of cancer, in a population of people who go on to die from cancer.

## Materials & methods

This was a retrospective cohort study of all 2,443 residents of Tayside, Scotland who died from cancer during a 30-month period to 2015. The cohort was identified posthumously using General Register Office death data, and people were included if they had ‘cancer’ in position 1 of the death certificate. The Community Health Index (CHI) number, a unique patient-identifying number used for all contacts in NHS Scotland, was used to link this cohort to demographic datasets. Demographic data at time of diagnosis were obtained from the Cancer Registry (Scottish Morbidity Records), Scottish Executive Urban Rural Classification (SEURC, which classifies postcodes in terms of remoteness and rurality), and Scottish Index of Multiple Deprivation (SIMD, which categorises deprivation into quintiles from SIMD 1 [most deprived] to SIMD 5 [least deprived]), and linked using CHI numbers. Data were analysed with SPSS v25, comparing cancer decedents who received late diagnoses versus those who did not. Binary logistic regression was used to evaluate associations between caner decedents’ timing of diagnosis and their demographic and clinical factors, including age, gender, rurality, deprivation and cancer type. Univariate and adjusted odds ratios with 95% confidence intervals (CIs) were calculated for each outcome. Full methodology detailed in STROBE Statement for Observational Studies (Appendix 1).

There is no agreed definition of ‘late diagnosis’ in cancer care, with previous publications suggesting definitions ranging from a few weeks to one year before death [7, 8, 23]. For the purposes of this study ‘late diagnosis’ was defined as a diagnosis of cancer within the last 12 weeks of life. Throughout this paper the term ‘cancer decedents’ is used to refer to people who went on to die from cancer.

## Results

### Timing of cancer diagnosis relative to death

**Fig. 1:**
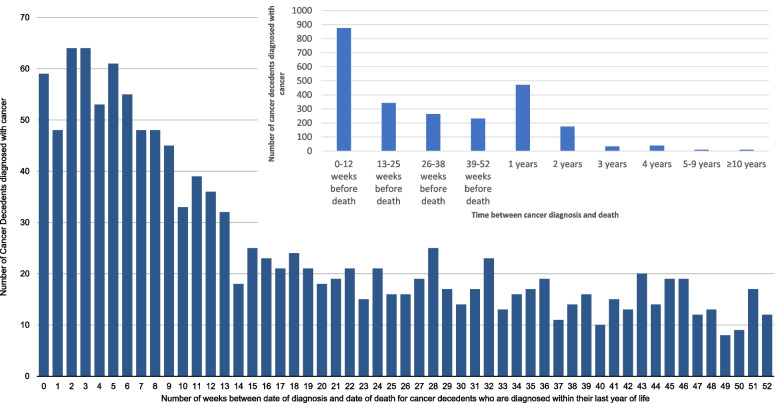
Time between diagnosis and death for cohort cancer decedents

This study demonstrated a substantial variation in the timing of diagnosis compared to death. One third of people (*n* = 831, 34.0%) received late diagnosis, being diagnosed within their last 12 weeks of life (Fig. 1).

### Patient-level factors associated with late diagnosis

The associations between age and cancer type and late diagnosis were seen on both univariate and multivariate analyses. Multivariate analysis used all other variables in the model for adjustment. Gender, rurality and deprivation showed no significant association with receiving a late diagnosis, either on univariate or multivariate analysis (Table 1).

**Table 1:** Logistic regression of demographic and clinical factors and associations with late diagnosis^a^

	Cohort of Cancer Decedents [*n* = 2,443 people (%)]	Cancer Decedents who received a Late Diagnosis^b^ [*n* = 831 people (%)]	Cancer Decedents who did Not received a Late diagnosis [*n* = 1,612 people (%)]	Univariate OR (95% CI)	Multivariate Adjusted OR (95% CI)
Age					
< 65 years	478	108 (22.6)	370 (77.4)	1	1
65–74 years	662	194 (29.3)	468 (70.7)	**1.43 (1.09 to 1.85)**	**1.41 (1.06 to 1.85)**
75–84 years	809	292 (36.1)	517 (63.9)	**1.92 (1.49 to 2.5)**	**2.04 (1.56 to 2.63)**
≥ 85 years	494	237 (48.0)	257 (52.0)	**3.13 (2.38 to 4.17)**	**3.45 (2.63 to 4.55)**
Gender					
Female	1,165	415 (35.6)	750 (64.4)	1	1
Male	1,278	416 (32.6)	862 (67.4)	0.87 (0.75 to 1.03)	0.84 (0.70 to 1.00)
Cancer type					
Lung	672	241 (35.9)	431 (64.1)	1	1
Upper GI	514	210 (40.9)	304 (59.1)	1.23 (0.97 to 1.56)	1.10 (0.85 to 1.41)
Bowel	303	92 (30.4)	211 (69.6)	0.78 (0.58 to 1.04)	**0.66 (0.49 to 0.89)**
Breast & Ovarian	237	41 (17.3)	196 (82.7)	**0.37 (0.26 to 0.54)**	**0.30 (0.20 to 0.44)**
Prostate	99	7 (7.1)	92 (92.9)	**0.14 (0.6 to 0.30)**	**0.11 (0.05 to 0.25)**
Haematological	241	101 (41.9)	140 (58.1)	1.28 (0.95 to 1.75)	1.08 (0.80 to 1.47)
Other	377	139 (36.9)	238 (63.1)	1.04 (0.81 to 1.35)	1.00 (0.76 to 1.32)
Rurality Grouped^c^					
Urban	1,588	549 (34.6)	1039 (65.4)	1	1
Accessible	587	204 (34.8)	383 (65.2)	1.01 (0.83 to 1.23)	1.11 (0.88 to 1.41)
Remote	235	71 (30.2)	164 (69.8)	0.82 (0.61 to 1.10)	0.83 (0.61 to 1.15)
Deprivation^d^					
SIMD5 1	469	145 (34.4)	277 (65.6)	1	1
SIMD5 2	528	139 (35.5)	253 (64.5)	1.05 (0.79 to 1.41)	0.96 (0.88 to 1.30)
SIMD5 3	447	163 (36.7)	281 (63.6)	1.11 (0.84 to 1.47)	1.00 (0.74 to 1.35)
SIMD5 4	495	237 (32.4)	494 (67.6)	0.92 (0.71 to 1.18)	0.84 (0.63 to 1.12)
SIMD5 5	471	140 (33.3)	281 (66.7)	0.95 (0.71 to 1.27)	0.87 (0.64 to 1.18)

^a^Significant results are indicated in bold font

^b^Diagnosed within the last 12 weeks of life

^c^‘Urban’ comprises SEUR1&2, ‘Accessible’ comprises SEUR3&5 and ‘Remote’ comprises SEUR 4 & 6. 33 people had missing information (excluded from this analysis)

^d^Scottish Index of Multiple Deprivation (SIMD). Category 1 is most deprived, and category 5 is least deprived. 33 people had missing information (excluded from analysis)

With respect to age, on univariate analysis, age (*p* < 0.001) and cancer type (*p* < 0.001) were significantly associated with having a late cancer diagnosis, compared to not having a late cancer diagnosis (Table 1). Cancer decedents aged > 85 years and those with lung cancer were most likely to receive late diagnoses. On multivariate analysis, compared to cancer decedents aged < 65 years, those aged 75–84 years had twice the odds of receiving a late diagnosis of cancer, and those aged ≥ 85 years were over three times more likely to receive a late cancer diagnosis.

With regard to cancer type, people with lung cancer were one and a half times more likely to have a late diagnosis than those with bowel cancer, three times more likely than those with breast and ovarian cancers, and nine times more likely than those with prostate cancer.

While gender was not statistically significant there was an observed tendency for women to be more likely to have a late diagnosis than men. Though it narrowly missed statistical significance, on multivariate analysis women were 16% more likely to receive a late diagnosis than men (95%CI OR0.84 (OR0.70 to 1.00).

## Discussion

These findings provide a useful guide to suggest which factors would confer the greatest benefit for targeted intervention. Importantly, age and cancer type were significantly associated with increased odds of receiving a late diagnosis. Gender, rurality and deprivation were not associated with odds of receiving a late diagnosis. Population interventions which focus on gender, rurality and deprivation, may therefore not have the desired impact on reducing late diagnosis. Resources for preventing late diagnosis may have a substantially greater impact if directed to interventions which target people with high-risk age and cancer type.

Our findings suggest that age is the single biggest predictor of late cancer diagnosis. Focusing on age-related barriers to accessing care would therefore be expected to have the greatest single-factor impact on reducing late diagnoses overall. Studies have shown that barriers to seeking medical help for symptoms of cancer include emotional, practical and service barriers [24], including difficulty making an appointment, and worry about wasting doctors’ time [25]. Such barriers to accessing care may be more predominantly experienced by older adults, compared to younger ones [26–28]. Previous population studies have suggested that adults over 65 years old have lower recall and recognition of warning signs of cancer, compared to younger adults [24, 25]. This relative lack of awareness regarding cancer, and barriers to accessing care, may cause delayed presentation to primary care in older people with cancer and result in cancers being diagnosed at a later stage [25].

The increased odds of receiving a late diagnosis among people with lung cancer, compared to other cancer types, suggests that in order to have the biggest impact on total number of late diagnoses and overall survivability, policy and public health interventions should prioritise the factors leading to delay diagnosis of lung cancer, above factors associated with other cancer types. The association between having lung cancer and receiving a late cancer diagnosis is multifactorial, and includes delayed identification of symptoms, delayed presentation to primary care, delayed referral to oncology or diagnostic testing, waiting times for investigations and review in secondary care [29, 30]. The significant association between having lung cancer and receiving a late diagnosis is particularly important, during the COVID-19 pandemic, in which there have been substantially fewer deaths attributed to lung cancer than would have been ordinarily expected [5]. This is likely due to cough being a cardinal symptom of covid, and people with undiagnosed lung cancer being more likely to die from COVID-19 than those without underlying cancer [16, 20].

### Comparison with existing literature

Most research surrounding late diagnoses in cancer has focused on specific cancer types, and has emphasised screening and diagnostic tests, rather than public awareness and clinical education [31–36]. The UK’s rates of late diagnosis are among the worse in Europe [6]. Late diagnosis can occur due to delays at any point in the diagnostic journey – including “‘patient delay’ (from onset of symptoms to their first presentation); ‘primary care delay’ (from first presentation in primary care to referral for further care or diagnostic investigation); ‘referral delay’ (from referral for further care or diagnostic investigation to being seen in secondary care); and ‘secondary care delay’ (from being first seen in secondary care to diagnosis) [37]”. While population-level factors that increase cancer risk are well-characterised in literature, there is no clear understanding of what patient-level factors contribute to receiving a late diagnosis of cancer on a population level [2, 23, 33, 37–39].

Most of the studies examining late diagnosis are small studies, reporting conflicting findings across variable healthcare settings, with different definitions of late diagnosis and with the potential confounder of lead-time bias [37, 39, 40]. Some evidence suggests that lung cancer men, older people, those living in rural areas, and in areas of high social deprivation are more likely to experience early mortality from cancer, though the impact of these factors on late diagnosis rather than early mortality, is unclear [29, 37, 41]. Previous research has suggested that, for colorectal cancer, younger people and people living in high areas of social deprivation were more likely to have late diagnoses, but found no impact on rurality [37, 42].

Papers in this field also tend to focus on cancer stage at time of diagnosis or impact of demographic factors on relative mortality risk [40]. Furthermore, the available literature is largely confined to studies exploring the impact of demographic factors on individual cancer types [40]. Publications reviewing the international literature related to late cancer diagnosis highlighted a paucity of research related to causes of late diagnosis across cancer types [40, 43]. Because this study examines a population cohort who have all died from cancer, it is possible to examine the impact of demographic factors on late diagnosis irrespective of cancer stage or mortality, and to do so in a way that corrects for cancer type. To the author’s knowledge, this is the first paper which has examined a range of demographic factors and corrected for cancer type in analysing the impact of demographic factors on receiving a late diagnosis. This paper is novel in that it uses cancer type as a covariate and analyses impact of other demographic factors adjusted for cancer type.

### Strengths and limitations

The completeness of the demographic and clinical datasets is a point of particular strength in this research. The demographic data on age, gender, cancer type, date of diagnosis, and date of death were entirely complete. The demographic data on rurality and deprivation were > 98.5% complete, with any missing information being unobtainable due to having postcodes with no corresponding SIMD and/or SEUR classification.

There were some limitations in terms of data availability. A limitation of the demographic data was the lack of information on ethnicity and smoking status, both of which are significantly associated with deprivation. Future analysis correcting for more covariates has the potential to identify new associations with late diagnosis that have not been possible to identify with the data available for this study.

Multiple social and psychological factors at person, carer and community levels, may affect late diagnosis, and would not have been apparent within the analysis undertaken in this research.

### Implications for research and/or practice

These findings suggest that public health and information campaigns aimed at increasing awareness of cancer symptoms, especially with regard to lung cancer, and encouraging earlier presentation by older people, and those with symptoms of lung cancer, may be the most effective methods of reducing avoidable late cancer diagnoses. Future research into how to overcome these barriers and mechanisms is needed in order to address this potential area of health inequality.

When caring for patients with confirmed or suspected malignancy, clinicians should have a heightened awareness that those with advanced age, or with symptoms suggestive of lung cancer are more likely to receive a late diagnosis, and should consider initiating onwards referral, anticipatory care planning and palliative care as soon as possible.

Identifying people who are at risk of receiving a late cancer diagnosis would allow physicians and policymakers to target resources and interventions at those people at greatest risk. This ensure the greatest impact of such interventions and facilitate effective anticipatory care planning for those with unavoidable late diagnoses, while maximising the efficacy of prevention strategies for avoidable late diagnoses.

## Conclusions

Older age and lung cancer were strongly associated with patients having increased odds of having a late cancer diagnosis, in a population of patients who went on to die from cancer. Practice and policies aimed at addressing those at higher risk of receiving a late cancer diagnosis could have greater impact if they focused on older people and those with lung cancer symptoms.

### Supplementary Information

**Additional file 1: Appendix 1.** STROBE Statement for Observational Studies: Methods

## Data Availability

The anonymised datasets that were generated and/or analysed during this study are not publicly available due to using deidentified but individual-level healthcare data. The data are accessible via the Health Informatics Centre, University of Dundee, and are available from the corresponding author upon reasonable request.

## References

[1] Cancer Research UK., Cancer Statistics for the UK. 2020 October 2021].

[2] Ferlay, J., et al., GLOBOCAN 2012 v1.0, Cancer incidence and mortality worldwide: IARC CancerBase No 11. Lyon: International Agency for Research on Cancer, 2013.

[3] Information Services Division (ISD) Report, Cancer Mortality in Scotland. A National Statistics Publication for Scotland, 2015.

[4] Cancer Research UK., Saving lives, averting costs: An analysis of the financial implications of achieving earlier diagnosis of colorectal, lung and ovarian cancer. 2014.

[5] Elliss-Brookes L (2012). Routes to diagnosis for cancer – determining the patient journey using multiple routine data sets. Br J Cancer.

[6] Berrino F., et al., EUROCARE Working group. Survival for eight major cancers and all cancers combined for European adults diagnosed in 1995–99: results of the EUROCARE-4 study. Lancet Oncol. 2007;8:773–783. 10.1016/S1470-2045(07)70245-0 17714991

[7] Hanna T P, K.W.D., Thibodeau S, Jalink M, Paulin G A, Harvey-Jones E et al. , Mortality due to cancer treatment delay: systematic review and meta-analysis BMJ, 2020. 371. 10.1136/bmj.m4087 PMC7610021 33148535

[8] Neal RD (2015). Is increased time to diagnosis and treatment in symptomatic cancer associated with poorer outcomes?. Systematic review British Journal of Cancer.

[9] Mills, S.E.E., et al., Community prescribing trends and prevalence in the last year of life, for people who die from cancer. BMC Palliative Care, 2022. 21(1). 10.1186/s12904-022-00996-3 PMC9264643 35799225

[10] Zhang B, Nilsson ME, Prigerson HG (2012). Factors important to patients’ quality of life at the end of life. Arch Intern Med.

[11] Gomes B (2012). Preferences for place of death if faced with advanced cancer: a popu- lation survey in England, Flanders, Germany, Italy, the Netherlands. Portugal and Spain Ann Oncol.

[12] Higginson I (2010). Time to get it right: are preferences for place of death more stable than we think?. Palliat Med.

[13] Henson L, Higginson I, Gao W (2018). What factors influence emergency department visits by patients with cancer at the end of life? Analysis of a 124,030 patient cohort. J Palliat Med.

[14] Wood, C. and J. Salter, A time and a place: what people want at the end of life. 2013.

[15] Dixon, J., et al., Equality in the Provision of Palliative Care in the UK: Review of Evidence. Marie Curie, 2015.

[16] Blaney J (2011). Hospital cancer deaths: late diagnosis and missed opportunity. BMJ Support Palliat Care.

[17] Mills SE, G.L., Buchanan D, et al Factors associated with unscheduled care use by cancer decedents: a systematic review with narrative synthesis BMJ Supportive & Palliative Care, 2020. 10.1136/bmjspcare-2020-002410 33051311

[18] Mills S (2019). Factors affecting use of unscheduled care for people with advanced cancer: a retrospective cohort study in Scotland. Br J Gen Pract.

[19] Mills , S., et al., Death from cancer: frequent unscheduled care. BMJ Support Palliat Care, 2022. 10.1136/bmjspcare-2021-003448 35351803

[20] Cancer Research UK. Evidence of the impact of COVID-19 across the cancer pathway: Key Stats. 2021 22 November 2021].

[21] Morris, E., et al., Impact of the COVID-19 pandemic on the detection and management of colorectal cancer in England: a population-based study. . Lancet, 2021. 10.1016/S2468-1253(21)00005-4 PMC7808901 33453763

[22] Maringe C (2020). The impact of the COVID-19 pandemic on cancer deaths due to delays in diagnosis in England, UK: a national, population-based, modelling study. Lancet Oncol.

[23] Weller D, Campbell C (2009). Uptake in cancer screening programmes: a priority in cancer control. Br J Cancer.

[24] Stubbings S (2009). Development of a measurement tool to assess public awareness of cancer. Br J Cancer.

[25] Robb K, Stubbings S, Ramirez A, Macleod U, Austoker J, Waller J, Hiom S, Wardle J (2009). Public awareness of cancer in Britain: a population-based survey of adults. Br J Cancer..

[26] Fitzpatrick AL, Powe NR, Cooper LS, Ives DG, Robbins JA (2004). Barriers to health care access among the elderly and who perceives them. Am J Public Health..

[27] Parliamentary and Health Services Ombudsman, Breaking down the barriers: Older people and complaints about health care. 2015: Millbank Tower Millbank, London, SW1P 4QP.

[28] Age UK, Improving Healthcare (England). May 2019, [https://www.ageuk.org.uk/globalassets/age-uk/documents/policy-positions/care-and-support/age-uk-improving-healthcare-policy-position.pdf Accessed 14 Jan 2023].

[29] Bjerager M, Palshof T, Dahl R, Vedsted P, Olesen F (2006). Delay in diagnosis of lung cancer in general practice. Br J Gen Pract..

[30] Ellis P, Vandermeer R (2011). Delays in the diagnosis of lung cancer. J Thorac Dis.

[31] Schiffman JD, F.P., Gibbs P. , Early detection of cancer: past, present, and future. Am Soc Clin Oncol Educ Book, 2915: p. 57–65. 10.14694/EdBook_AM.2015.35.57 25993143

[32] Necula L, Matei L, Dragu D, Neagu AI, Mambet C, Nedeianu S, Bleotu C, Diaconu CC, Chivu-Economescu M (2019). Recent advances in gastric cancer early diagnosis. World J Gastroenterol..

[33] Shamsi M, Islamian PJ (2017). Breast cancer: early diagnosis and effective treatment by drug delivery tracing. Nucl Med Rev Cent East Eur.

[34] Kessler T (2017). Cervical Cancer: Prevention and Early Detection. Semin Oncol Nurs.

[35] Sullivan FM (2021). Earlier diagnosis of lung cancer in a randomised trial of an autoantibody blood test followed by imaging. Eur Respir J.

[36] Adams D (2014). Oral cancer: early diagnosis. Dent Today.

[37] Allgar VL, Neal RD (2005). Delays in the diagnosis of six cancers: analysis of data from the National Survey of NHS Patients: Cancer. Br J Cancer.

[38] White MC (2014). Age and Cancer Risk. Am J Prev Med.

[39] Laudicella M (2018). What is the impact of rerouting a cancer diagnosis from emergency presentation to GP referral on resource use and survival? Evidence from a population-based study. BMC Cancer.

[40] Richards MA (2009). The National Awareness and Early Diagnosis Initiative in England: assembling the evidence. Br J Cancer.

[41] Tracey E (2015). Survival of Australian lung cancer patients and the impact of distance from and attendance at a thoracic specialist centre: a data linkage study. Thorax.

[42] Blair A, Datta GD (2020). Associations between area-level deprivation, rural residence, physician density, screening policy and late-stage colorectal cancer in Canada. Cancer Epidemiol.

[43] Austoker J, B.C., Forbes LJL, Atkins L, Martin F, Robb K, Wardle J, Ramirez AJ,  (2009). Interventions to promote cancer awareness and early presentation: systematic review. Br J Cancer.

