# Supplementary material for: Age and cancer type: associations with increased odds of receiving a late diagnosis in people with advanced cancer
Source: BMC Cancer. 2023 Nov 30;23:1174. doi: 10.1186/s12885-023-11652-1 (PMC10691149; doi:10.1186/s12885-023-11652-1)
Supplement: Supplementary file 1 — Additional file 1: Appendix 1. STROBE Statement for Observational Studies: Methods [file 12885_2023_11652_MOESM1_ESM.docx]

## **APPENDIX 1: STROBE Statement for Observational Studies: Methods**

**Study design**

Observational retrospective cohort study.

**Setting**

Set in NHS Tayside, Scotland, in a 30-month period from 2013-2015.

**Participants**

This was a retrospective cohort study of all 2,443 residents of the Tayside region of Scotland (total population = approximately 410,000^1^) who died from cancer between 01/03/2013 and 30/06/2015 . The study population was identified posthumously using General Register Office death registration data. Individuals were included in the cohort if their cause of death was cancer-related, which, for the purposes of this study, was defined as having a cancer code in part I of the cause of death section of their death certificate. The cohort population was limited to adults as, in NHS Tayside, children with cancer are given direct access information for paediatrics, who arrange direct review admissions for any unscheduled care contacts

**Variables**

Data linkage was done using CHI numbers to link the study cohort to datasets held by in the Health Informatics Centre (HIC). The datasets that were linked to the CHI cohort were as follows, with each dataset showing the breakdown of its data components:

- Demography: PRO-Chi, Sex, Anonymised date of birth, Date of moving to NHS Tayside (if relevant), Date of moving out of NHS Tayside (if relevant), SIMD5, SIMD10, Anonymised practice code, SEUR06 (rurality), Anonymised postcode
- Cancer Registry (SMR06): PRO-Chi, Incidence date, Site, ICD10 classification, Date of diagnosis, Cause of Death (Cause 1, Cause 2, Cause 3, Cause 4), Grade, Stage, Chemotherapy (whether or not patients had received chemotherapy), Health Board region, SIMD5 and SIMD10
- General Registry Office (GRO) Death Data: Date of death, Cause of Death (Cause 1, Cause 2, Cause 3, Cause 4), Date of registration of death, Year of registration of death, InstCode (location at which the health activity took place), Duration of illness in days, Duration of illness in months, Duration of illness in years,
- CHI Death Data: PRO-Chi, Date of Death, Source of death information

Cancer codes were identified using International Statistical Classification of Diseases and Related Health Problems – 10^th^  Revision (ICD10) 2016^2^. The ICD10 classifications for Neoplasms (C00-C97X) were used to identify deaths as being from cancer.

**Data sources/ measurement**

Routinely collected clinical data for all attendances in the last year of life was linked using the Community Health Index (CHI) number, which is used as a single patient identifier throughout NHS Scotland. CHI-linked data were obtained from the Cancer Registry (Scottish Morbidity Records), Scottish Executive Urban Rural Classification (SEURC classifies postcodes in terms of remoteness and rurality), Scottish Index of Multiple Deprivation [SIMD, categorises deprivation into quintiles from SIMD1 (most deprived) to SIMD5 (least deprived)^3^].

**Bias**

***Study size***

This study included all 2,443 residents of NHS Tayside who died from cancer during the study time period. The sample size calculation shows that with 2,443 people in the cohort this study has 90% power in a logistic regression model to be able to detect odds ratios from 1.15 or above at the 5% significance level with a multiple correlation coefficient of 0.3 using the method of Hsieh^4^.

***Quantitative variables***

#### Age

Participants’ age was taken at their time of death. This is a ‘calculated age’ and is not accurate but is close to the patient’s actual age. Actual age is potentially patient-identifiable.

####

#### Deprivation

The Scottish Index of Multiple Deprivation (SIMD) an official statistical tool used by the Scottish Government's to identify, classify and describe areas of multiple deprivation in Scotland^5^. The SIMD ranks small areas, referred to as Data Zones, into categories to describe their level of deprivation^5^. SIMD takes into account current income, employment, health, education, skills and training, housing, geographic access and crime, and generates a numerical score to reflect an area’s level of deprivation. SIMD1 reflects areas with the most deprivation and SIMD5 describes areas that are least deprived^5^.

SIMD ranks can be sorted into quintiles (which splits the data into 5 groups each containing 20% of Scotland’s data zones), deciles (10 groups each containing 10% of data zones in Scotland) and vigintiles (20 groups each containing 20% of data zones in Scotland), and are based on Data Zones from the 2011 census^5^. For the purposes of this research the quintiles – SIMD5 – were used.

#### Rurality

The rurality of the people in the cohort was recorded in the demography file using the SEUR6 variable. SEUR6 stands for the Scottish Urban Rural Classification 6 classification system. The SEUR system defines settlements of 3,000 or less people to be rural and determines whether areas are remote based on drive times from settlements of 10,000 or more people. It uses six categories to describe rurality. In order to map rurality more effectively against unscheduled care usage, rurality was ‘grouped’ into three distinct categories which were based on the level of accessibility. For each of these groups two SEUR code were paired, in the following combinations: 1. Urban (SEUR codes 1&2), Accessible (SEUR Codes 3&5) and Remote (SEUR 4&6).

### Statistical Methods

Data were cleaned, anonymised and stored in the Safe Haven platform in the Health Informatics Centre (HIC). Safe Haven is a remote-access virtual environment that exists to protect data confidentiality, protect data from data loss or misuse, and have secure management and processing of data which is overseen by trained Data Controllers. In the patient-level analysis, binary logistic regression and multivariate logistic regression were used in order to examine associations between late diagnoses and demographic and clinical factors. Univariate and adjusted odds ratios with their 95% confidence intervals (CIs) were calculated for each outcome. All analyses were conducted using SPSS v25.

#### Missing data:

#### Deprivation and Rurality

Of the 2,443 in the cohort, 33 had missing data for both rurality (SEUR) and deprivation (SIMD) which is a relatively small proportion and so could be considered missing completely at random (MCAR). This ‘missingness’ was due to these people having postcodes which were not represented in the SEUR and SIMD classifications. In order to determine how to treat the missing data, a comparison of people with complete data and those with missing data was done. On Chi-squared testing there was no significant difference between those with missing data and those with complete data for any demographic factors, including age (p=0.420), gender (p=0.337), cancer type (p=0.585) or timing of diagnosis by quintile(p=0.136) or within/not within the last year of life (p=0.464). These was also no significant difference between those with missing data and complete data in terms of use or non-use of unscheduled care (p=0.467), total number of unscheduled care attendances (p=0.474), number of GPOOH appointments (p=0.996) and number of A&E appointments (p=0.785) in the last year of life. There was no difference between those with missing and complete data in likelihood of attending GPOOH (p=0.710) or A&E (p=0.227). Had there been any significant differences found the missing data would have been accounted for using multiple imputation; however, because there were no significant differences in any of the demographic factors, cancer types and timings of diagnosis, the people with missing SEUR and SIMD data were excluded by default when running analyses and so assumed to be missing completely at random.

#### Missing Data: Cancer Type

There were only 6 people out of the 2,443 for whom there was no entry on the cancer registry (SMR06). These people were identified for the cohort based on General Registry Office (GRO) death data (cancer in position 1). It is unclear why these people were not included on the cancer registry, but possibilities include that they were diagnosed very close to death or that they were diagnosed elsewhere (e.g. in England) and were not seen by oncology during their time in NHS Tayside. For these patients, the ‘cancer type’ was taken to be the cancer listed in position 1 on their death certificate. There was complete GRO death data for each of the 6 people not on the cancer registry, meaning that the ‘cancer type’ information was complete for all 2,443 cohort participants when extracted from both the cancer registry and GRO information.

**Appendix 1 References:**

1. Donnan, P., Dorward, DWT., Mutch, W. & Morris, AD. Development and Validation of a Model for Predicting Emergency Admissions Over the Next Year (PEONY). Jama 168 (2008).

2. World Health Organisation. Vol. 10th Revision (2016).

3. Scottish Government. SIMD Frequently Asked Questions, Accessed from 20 February 2020: <https://www2.gov.scot/Topics/Statistics/SIMD/FAQUsingSIMD> (2013).

4. Hsieh, F. Y. Sample size tables for logistic regression. . Statist. Med 8, 795–802 (1989).

5. Scottish Government. Introducing The Scottish Index of Multiple Deprivation. (2016).
